# Gender differences in the perception of asthma and respiratory symptoms in a population sample of asthma patients in four Brazilian cities*

**DOI:** 10.1590/S1806-37132014000600002

**Published:** 2014

**Authors:** Laura Russo Zillmer, Mariana Rodrigues Gazzotti, Oliver Augusto Nascimento, Federico Montealegre, James Fish, José Roberto Jardim

**Affiliations:** Federal University of São Paulo, Paulista School of Medicine, São Paulo, Brazil. Pulmonary Rehabilitation Center, Federal University of São Paulo Paulista School of Medicine, São Paulo, Brazil; Federal University of São Paulo, Paulista School of Medicine, São Paulo, Brazil. Pulmonary Rehabilitation Center, Federal University of São Paulo Paulista School of Medicine, São Paulo, Brazil; Federal University of São Paulo, Paulista School of Medicine, São Paulo, Brazil. Federal University of São Paulo Paulista School of Medicine, São Paulo, Brazil; University of Puerto Rico, School of Public Health, Reio Piedras, PR, USA. Merck, Sharp & Dohme Corp., Carolina, PR, USA; and Professor. University of Puerto Rico School of Public Health, Reio Piedras, PR, USA; Merck Sharp & Dohme Corp., Whitehouse Station, NJ, USA. Merck Sharp & Dohme Corp., Whitehouse Station, NJ, USA; Federal University of São Paulo Paulista, School of Medicine, São Paulo, Brazil. Federal University of São Paulo Paulista School of Medicine, São Paulo, Brazil

**Keywords:** Asthma/diagnosis, Asthma/prevention & control, Quality of life

## Abstract

**OBJECTIVE:**

To evaluate the impact of asthma, by gender, in a population sample of asthma patients in Brazil.

**METHODS:**

We conducted face-to-face interviews with 400 subjects (> 12 years of age) included in a national probability telephone sample of asthma patients in the Brazilian state capitals of São Paulo, Rio de Janeiro, Curitiba, and Salvador. Each of those 400 subjects completed a 53-item questionnaire that addressed five asthma domains: symptoms; impact of asthma on quality of life; perception of asthma control; exacerbations; and treatment/medication.

**RESULTS:**

Of the 400 patients interviewed, 272 (68%) were female. In relation to respiratory symptoms, the proportion of women reporting extremely bothersome symptoms (cough with sputum, tightness in the chest, cough/shortness of breath/tightness in the chest during exercise, nocturnal shortness of breath, and nocturnal cough) was greater than was that of men. Daytime symptoms, such as cough, shortness of breath, wheezing, and tightness in the chest, were more common among women than among men. Women also more often reported that their asthma interfered with normal physical exertion, social activities, sleep, and life in general. Regarding the impact of asthma on quality of life, the proportion of subjects who reported that asthma caused them to feel that they had no control over their lives and affected the way that they felt about themselves was also greater among women than among men.

**CONCLUSIONS:**

Among women, asthma tends to be more symptomatic, as well as having a more pronounced effect on activities of daily living and on quality of life.

## Abstract (pt)

**OBJETIVO:**

Avaliar o impacto da asma em relação ao sexo em uma amostra populacional de pacientes asmáticos no Brasil.

**MÉTODOS:**

Foram entrevistados pessoalmente 400 pacientes asmáticos com idade > 12 anos de uma amostra probabilística nacional por contato telefônico nas cidades de São Paulo, Rio de Janeiro, Curitiba e Salvador. Os indivíduos responderam um questionário de 53 questões relacionadas com cinco domínios da asma: sintomas; impacto da asma na vida; percepção do controle da asma; exacerbações; tratamento e medicação.

**RESULTADOS:**

Dos 400 pacientes entrevistados, 272 (68%) eram do sexo feminino. Em relação aos sintomas respiratórios, uma maior proporção de mulheres relatou se sentir extremamente incomodada com seus sintomas (tosse com secreção, sensação de aperto no peito, tosse/falta de ar/sensação de aperto no peito durante exercícios, falta de ar noturna e tosse noturna) do que os homens. Sintomas diurnos, como tosse, falta de ar, chiado e sensação de aperto no peito, foram mais comuns nas mulheres que nos homens. Além disso, a asma interferiu mais frequentemente nos esforços físicos normais, atividades sociais, durante o sono e na vida em geral nas mulheres. Sobre o impacto da asma na qualidade de vida, as mulheres relataram mais frequentemente que os homens que a asma causava uma sensação de falta de controle sobre a própria vida e que eram afetadas na forma como se sentiam em relação a si mesmas.

**CONCLUSÕES:**

As mulheres asmáticas apresentam mais sintomas e são mais afetadas em suas atividades diárias e qualidade de vida.

## Introduction

Bronchial asthma is a chronic inflammatory disease characterized by airflow limitation that resolves spontaneously or with treatment. This inflammation also causes increased airway responsiveness. ^(^ 1 ^)^ These pathophysiological changes cause recurrent episodes of wheezing, dyspnea, tightness in the chest, and cough, particularly at night and upon waking in the morning.^(^ 2 ^)^

Asthma is one of the most prevalent chronic diseases worldwide and is the most common indication for hospitalization among children. ^(^ 3 ^)^ In Brazil, the prevalence of asthma among children and adolescents is close to 20% and is high in all regions of the country.^(^ 2 ^)^ Environmental factors, including allergens, cigarette smoke, air pollution, and viral respiratory infections, are associated with asthma symptoms and severity. Some factors, such as family history, atopy, and gender, also play a role in the development and progression of asthma.^(^ 4 ^)^

Current epidemiological studies suggest that there is a higher prevalence of asthma among males before puberty, after which asthma predominates among females.^(^ 5 ^)^ Women experience greater increases in the prevalence of and mortality from asthma over time,^(^ 6 ^)^ have a greater prevalence of bronchial hyperresponsiveness,^(^ 7 ^)^ and use health care services more often,^(^ 8 ^)^ including visits to the emergency room and hospitalizations,^(^ 9 ^)^ as well as reporting respiratory symptoms more often^(^ 10 ^)^ and poorer quality of life.^(^ 11 ^,^ 12 ^)^ Because asthma is a typical multifactorial disease, in which genetic, environmental, pathophysiological, and immunological factors play a role, the reasons for specific gender differences may also be multiple, such as differences in airway physiology and pathology, hormonal interference in women,^(^ 13 ^)^ differences in immune response to high-titer measles vaccine,^(^ 14 ^)^ risk of infectious diseases in childhood,^(^ 15 ^)^ and behavioral differences between men and women.^(^ 16 ^)^

In Brazil, the prevalence of asthma^(^ 17 ^)^ and wheezing^(^ 18 ^)^ has been shown to be higher in two age groups of boys: 6-7 years and 10-12 years. ^(^ 19 ^)^ In two other studies conducted in Brazil, girls aged 13-14 years had more respiratory symptoms than did boys of the same age.^(^ 20 ^,^ 21 ^)^ However, there are still few studies investigating the frequency of attacks, the impact of asthma, and the intensity of symptoms that affect men and women differently. Therefore, the objective of the present study was to evaluate, in a population sample of asthma patients in Brazil, the possibility of gender differences in five asthma domains: symptoms; impact of asthma on quality of life; perception of asthma control; exacerbations; and treatment/medication. This information will enhance the existing knowledge of how asthma affects men and women and can be useful for developing asthma management plans.

## Methods

The Latin America Asthma Insight and Management survey was conducted in five Latin American countries (Argentina, Brazil, Mexico, Venezuela, and Puerto Rico) and followed the same design as that of the Asthma Insight and Management surveys conducted in the United States, Europe, Canada, Asia, and the Pacific region. The objective of the survey was to investigate asthma patients' perception of their disease.^(^ 22 ^)^

**Figure 1 -:**
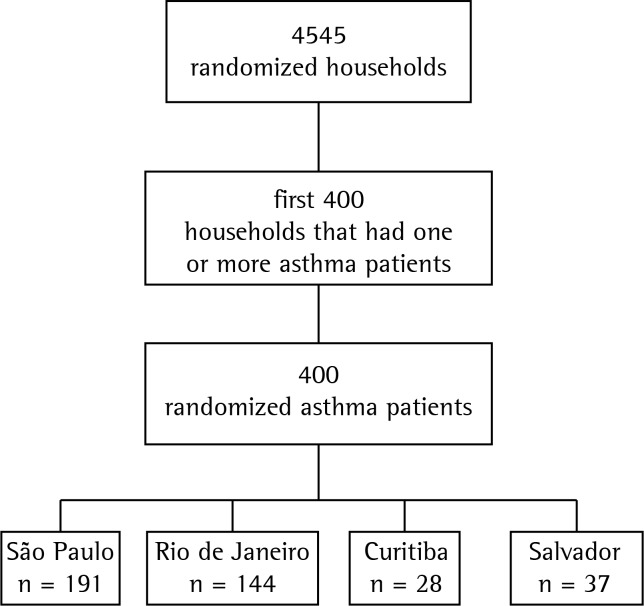
Flowchart of patients eligible for the study.

In Brazil, 4,545 households were selected from a national probability sample in four cities (São Paulo; Rio de Janeiro; Curitiba; and Salvador). The selected households were contacted by phone to investigate whether there were any subjects with physician-diagnosed asthma there. If there were two or more subjects with asthma in the household, only one of them was randomly selected for a scheduled face-to-face interview, which was conducted by trained professional interviewers. In total, 400 patients or parents/legal guardians of those aged 12-17 years were interviewed face to face (Figure 1). The questionnaire consisted of 53 questions concerning five major asthma domains: symptoms; impact of asthma on quality of life; perception of asthma control; exacerbations; and treatment/medication. Each interview lasted approximately 35 minutes.

Each domain addressed by the questionnaire was assessed at two time periods: the past 12 months and the past four weeks. For the past 12 months, history of exacerbations, symptoms of worsening disease, severe attacks, hospitalization, emergency room visits, and regular medical visits were assessed based on the recollection of respondents. In addition, still regarding the past year, the impact of asthma on patients' lives was assessed by using questions concerning the frequency of asthma-related absences from school or work, as well as asthma-related limitation in activities of daily living, productivity on days when experiencing an asthma attack, and the influence of the disease on quality of life. Activities of daily living were assessed by five questions concerning sports, recreation, physical exertion, social activities, sleep, and interference with life. Respondents scored each question on a 1-4 point scale, corresponding to the degree of asthma-related limitation in those activities (1: high interference; 2: some interference; 3: very little interference; and 4: no interference).

The frequency of daytime and nocturnal respiratory symptoms, its relationship with usual activities, and the use of rescue and maintenance medication were assessed for the past four weeks. On the basis of these assessments, patients were classified as having well-controlled asthma, partially controlled asthma, or uncontrolled asthma, as defined by the Global Initiative for Asthma (GINA). ^(^ 22 ^)^ Those same parameters were also assessed for the worst month in the past 12 months.

The study was approved by the Research Ethics Committee of the Federal University of São Paulo *Hospital São Paulo* (CEP no. 238487 of April 5, 2013).

### Statistical analysis

Numerical data are presented as mean and standard deviation, whereas categorical data are expressed as frequency and proportion. The chi-square test was used to compare categorical data between the groups (males and females), and the Student's t-test was used to compare the means. The frequency of respiratory symptoms was rated from 1 to 4, with 1 and 4 meaning "every day" and "never", respectively. The impact of asthma on sports, normal physical exertion, social activities, sleep, and life in general was scored from 1 to 4, with 1 and 4 meaning "asthma interfered a lot" and "asthma did not interfere at all", respectively. Values of p < 0.05 were considered statistically significant.

## Results

We interviewed 400 asthma patients in four Brazilian cities: 191 in São Paulo (47.8%); 144 in Rio de Janeiro (36.0%); 28 in Curitiba (7.0%); and 37 in Salvador (9.2%). Of those patients, 128 (32%) were male and 272 (68%) were female. There were no gender differences in age, smoking status, level of education, number of asthma patients in the household, passive smoking, presence of pets in the household, or adherence to maintenance medication in the past four weeks (Table 1). In relation to asthma control, as defined by GINA criteria, the gender proportion in the controlled asthma group (10.2% men vs. 8.8% women) and in the partially controlled asthma group (64.8% men vs. 52.6% women) was similar; however, a greater proportion of females was observed in the uncontrolled asthma group (25.0% men vs. 38.6% women; p = 0.02). We found no statistically significant gender difference for use of maintenance medication in the past four weeks (p = 0.56).

**Table 1 -:** Demographic data of the 400 asthma patients interviewed in Brazil.a

Data	Men	Women	p
	(n = 128)	(n = 272)	
Age group			
12-17 years (n = 42)	13.8 ± 2.8	13.5 ± 2.2	0.67
18-40 years (n = 183)	28.7 ± 6.5	29.7 ± 6.2	
> 40 years (n = 175)	53.8 ± 11.1	53.0 ± 9.8	
Smoking status			
Smoker	32 (25.0)	64 (23.5)	0.15
Former smoker	39 (30.5)	64 (23.5)	
Nonsmoker	56 (43.8)	143 (52.9)	
Level of education			
Illiterate/ < 4 years of schooling	21 (16.4)	40 (14.8)	0.60
≤ 9 years of schooling	22 (17.2)	36 (13.3)	
High school	66 (51.6)	158 (58.3)	
College	19 (14.8)	37 (13.7)	
Number of asthma patients in the household	1.13 ± 0.36	1.24 ± 0.57	0.06
Passive smoker as a child	64 (50.0)	139 (51.1)	0.49
Passive smoker at home	47 (37.0)	90 (33.3)	0.13
Pets	63 (50.0)	136 (50.0)	0.33

a Values expressed as n (%) or mean ± SD.

Table 2 shows that the proportion of women reporting cough with sputum, tightness in the chest, cough/shortness of breath/tightness in the chest during exercise, and nocturnal symptoms (shortness of breath and cough) was greater than was that of men (p < 0.05). Women reported a greater frequency of these symptoms than did men (Table 3).

**Table 2 -:** Frequency and proportion of asthma patients who reported extremely bothersome respiratory symptoms in the past four weeks, by gender.

Symptoms	Men	Women	p
	(n = 128)	(n = 272)	
Daytime cough	37 (29.1)	98 (36.7)	0,14
Daytime shortness of breath	48 (38.4)	116 (44.1)	0,43
Breathlessness/wheezing	49 (39.2)	129 (48.5)	0,23
Cough with sputum	34 (27.2)	92 (36.7)	0,01
Tightness in the chest	34 (28.3)	116 (46.0)	0,005
Cough/shortness of breath/tightness in the chest during exercise	40 (33.3)	110 (43.8)	0,03
Waking at night with shortness of breath	54 (45.8)	144 (57.6)	0,01
Waking at night with a cough	51 (41.1)	138 (55.0)	0,002

a Values expressed as n (%).

**Table 3 -:** Respiratory symptom score during the worst month in the past 12 months, by gender (1: every day; 4: never).

Symptoms	Men	Women	p
	(n = 128)	(n = 272)	
Daytime cough	2.38 ± 0.68	2.21 ± 0.74	0.03
Daytime shortness of breath	2.77 ± 0.63	2.56 ± 0.78	0.007
Breathlessness/wheezing	2.59 ± 0.68	2.39 ± 0.84	0.02
Cough with sputum	2.71 ± 0.70	2.60 ± 0.88	0.19
Tightness in the chest	2.74 ± 0.69	2.51 ± 0.87	0.01
Cough/shortness of breath/tightness in the chest during exercise	2.73 ± 0.76	2.66 ± 0.89	0.47
Nocturnal shortness of breath	2.58 ± 0.82	2.48 ± 1.03	0.33
Nocturnal cough	2.48 ± 0.85	2.44 ± 0.99	0.72

a Values expressed as mean ± SD.

In comparison with men, women more often reported that their asthma interfered with normal physical exertion (p = 0.005), social activities (p = 0.001), sleep (p = 0.006), and life in general(p = 0.01), but not for sports/recreation (Table 4). The proportion of women who agreed with the statements "asthma causes me to feel that I have no control over my life" and "asthma affects the way I feel about myself" was significantly greater than was that of men (Table 5).

**Table 4 -:** Asthma-related limitation in activities of daily living, as determined by the score (1: high interference; 4: no interference), by gender.a

Activities	Men	Women	p
	(n = 128)	(n = 272)	
Sports/recreation	2.62 ± 1.04	2.63 ± 1.08	0.93
Normal physical exertion	2.74 ± 1.05	2.43 ± 1.01	0.005
Social activities	3.27 ± 0.94	2.89 ± 1.05	0.001
Sleep	2.54 ± 1.05	2.22 ± 1.07	0.006
Interference with life	2.95 ± 0.98	2.65 ± 1.10	0.01

a Values expressed as mean ± SD.

**Table 5 -:** Frequency and proportion of asthma patients who agreed with any of the statements below, by gender.a

Statements	Men	Women	p
	(n = 128)	(n = 272)	
Asthma causes me to feel that I have no control over my life.	50 (39.1)	141 (52.0)	0.02
I feel incapable because of asthma, compared with my friends.	33 (25.8)	81 (29.8)	0.52
Asthma affects the way I feel about myself.	49 (38.3)	147 (54.0)	0.01
At least once in my lifetime, I have had such a severe asthma episode that I thought my life was at risk.	37 (28.9)	100 (36.8)	0.07

a Values expressed as n (%).

## Discussion

Our results suggest that, compared with men with asthma, women with asthma experience a greater impact and frequency of respiratory symptoms and more often have asthma-related limitations in daily living. These differences have been the target of many studies, which investigate physiological, psychological, and environmental mechanisms related to asthma. In contrast to the literature, we found no gender differences in the prevalence of asthma even after stratification into different age groups.

It is known that, up to the age of 10-12 years, asthma is more prevalent among boys than among girls, and that, after puberty, it becomes more common among girls. It was not possible to assess age-related trends in asthma prevalence, because the sample consisted of individuals aged over 12 years.

The higher prevalence of asthma among women during their reproductive years coincides with increased bronchial hyperresponsiveness.^(^ 23 ^)^ Approximately 20-40% of women with asthma report worsening of their respiratory symptoms during the premenstrual and menstrual period. ^(^ 24 ^,^ 25 ^)^ The mechanism of worsening of asthma during the menstrual cycle remains unknown, but suggestions include increased serum levels of progesterone,^(^ 26 ^)^ increased mucous secretions,^(^ 27 ^)^ increased synthesis of prostaglandins during the premenstrual period,^(^ 28 ^)^ and abnormal β_2_-adrenergic receptor regulation. ^(^ 27 ^)^ Although the effect of endogenous estrogen on the airways has yet to be clearly defined, exogenous hormones can also influence asthma in women.^(^ 29 ^)^ The hormonal factor seems to be a key component to understanding the differences observed between genders.

Compared with men, women reported experiencing a greater impact of symptoms of cough with sputum, tightness in the chest, and cough/shortness of breath/tightness in the chest during exercise, as well as of nocturnal symptoms (shortness of breath and cough). In addition to feeling more troubled, women more often had daytime cough and shortness of breath and became breathless, with wheezing and tightness in the chest, than did men. These results were similar to those found in other studies, in which women had a higher frequency of respiratory symptoms, such as cough and wheezing.^(^ 30 ^,^ 31 ^)^ One group of authors demonstrated a relationship between greater perception of shortness of breath and female gender,^(^ 10 ^)^ whereas another study showed that perception of dyspnea was dependent on bronchial responsiveness rather than on gender.^(^ 32 ^)^ Studies have shown that symptoms in men and women can be inherently different, representing a difference in the underlying mechanism of the disease or a difference in the perception of the symptom. Kauffmann & Becklake showed that, for any given FEV_1_ value, women experienced a greater degree of shortness of breath than did men.^(^ 33 ^)^ In addition, women had a more sensitive cough reflex to a given stimulus.^(^ 34 ^)^ On the basis of this information, some authors have argued that, compared with men, women have a greater perception of respiratory symptoms, especially shortness of breath.

Regarding activities of daily living, women experienced greater asthma-related limitation in normal physical exertion, social activities, and sleep, as well as higher interference with life. Given that they have a greater perception and frequency of respiratory symptoms, it is easy to understand that they develop more limitations in activities of daily living. These aspects directly affect the quality of life of women with asthma, explaining the fact that, compared with men with asthma, they have poorer quality of life, as described in previous studies.^(^ 8 ^,^ 12 ^,^ 35 ^)^

The proportion of women who agreed that asthma caused them to feel that they had no control over their lives and that the disease affected the way they felt about themselves was also found to be greater. These results reflect how asthma reduces self-esteem in women with asthma. According to the results of a study, the correlation between dyspnea and the tendency toward social desirability is negative in men but positive in women.^(^ 11 ^)^ Psychologically, this could be explained by a certain repression. For men, being sick can be limiting in terms of social and professional relationships, and therefore, they tend to ignore their disease, especially in terms of social desirability. In contrast, for women, the disease may lead them to seek social support, which is reflected by a greater tendency toward social desirability. This interpretation is obviously only speculation, but it can explain the results obtained.

Quality of life impairment among women is not a characteristic only of asthma, but also of other chronic diseases.^(^ 12 ^,^ 36 ^)^ It has been suggested that the relationship between gender and quality of life in asthma is confounded by the higher levels of anxiety and depression among women. ^(^ 35 ^)^ One group of authors found that, despite having lower mortality and higher life expectancy, women with asthma reported poorer health-related quality of life compared with men of the same age with asthma.^(^ 12 ^)^

One of the explanations for the difference found between genders could be the greater exposure of women to risk factors, such as household environmental factors and presence of pets in the household, as well as less use of maintenance medication. However, in the present study, there were no significant gender differences in exposure to passive smoking as a child or at home, presence of pets, or use of maintenance medication. Nevertheless, even experiencing similar exposures, men and women can show different patterns of response to allergen exposure.^(^ 37 ^)^ Elderly males and females exposed to air pollution and showing high total IgE levels and airway hyperreactivity have been reported to have different responses to the same level of exposure: whereas women had a decrease in PEF in the morning, men were more likely to have symptoms such as cough.^(^ 37 ^)^ Although we found no significant differences for those variables, other forms of environmental exposure that were not investigated in the present study, such as exposure to gas used for cooking, may contribute to gender differences.^(^ 4 ^,^ 38 ^)^

Another relevant finding was the great proportion of patients (36.8% of women and 28.9% of men) who reported that, at least once in their lifetime, they had had such a severe asthma episode that they thought their lives were at risk. The proportion of women who reported this feeling was greater than that of men, but the difference was not significant. Attention should be drawn to the fact that most asthma attacks can be considered preventable, since there is effective drug treatment for asthma and there are self-management plans for asthma control.^(^ 1 ^)^

It is of paramount importance that health care professionals be cognizant of and understand the greater impairment in women than in men with asthma. Appropriate asthma management requires an ongoing partnership between the patient and her physician regarding physiological factors (e.g., sexual hormones, obesity, pregnancy, and depression) and non-physiological factors (e.g., smoking and drug adherence), all of which can contribute to improving asthma control. Asthma-related risks seem to be higher for women than for men, but the implementation of a specific management plan, including psychological support, could certainly help reduce the burden of asthma on women.

The present study has some limitations that are inherent in epidemiological studies. The sample for the study was selected via a probability sample and an initial telephone contact, in four Brazilian state capitals. Another limitation could be the fact that data were collected on the basis of information provided by patients rather than on information abstracted from medical records. However, the present study differs from other epidemiological studies, because asthma patients were defined only as those who had a physician-confirmed diagnosis of asthma. The second difference was the fact that all interviews were conducted face to face. In addition, the results found here are consistent with those found in the literature.

In conclusion, to our knowledge, this is the first study in Brazil that has shown clearly and in depth that, among women, asthma is more poorly controlled, causes greater interference with life, and is associated with a higher frequency of respiratory symptoms. These factors result in women experiencing poorer quality of life and greater social distress compared with men. Our study provides solid support for the clinical importance of physical and psychological differences between genders, as well as underscoring the fact that there is a need to implement gender-specific approaches to asthma management. Future studies are needed to investigate gender differences in the development and progression of asthma.
